# The *ets*-Related Transcription Factor GABP Directs Bidirectional Transcription

**DOI:** 10.1371/journal.pgen.0030208

**Published:** 2007-11-16

**Authors:** Patrick J Collins, Yuya Kobayashi, Loan Nguyen, Nathan D Trinklein, Richard M Myers

**Affiliations:** Department of Genetics, Stanford University School of Medicine, Stanford, California, United States of America; Lawrence Livermore National Laboratory, United States of America

## Abstract

Approximately 10% of genes in the human genome are distributed such that their transcription start sites are located less than 1 kb apart on opposite strands. These divergent gene pairs have a single intergenic segment of DNA, which in some cases appears to share regulatory elements, but it is unclear whether these regions represent functional bidirectional promoters or two overlapping promoters. A recent study showed that divergent promoters are enriched for consensus binding sequences of a small group of transcription factors, including the ubiquitous *ets*-family transcription factor GA-binding protein (GABP). Here we show that GABP binds to more than 80% of divergent promoters in at least one cell type. Furthermore, we demonstrate that GABP binding is correlated and associated with bidirectional transcriptional activity in a luciferase transfection assay. In addition, we find that the addition of a strict consensus GABP site into a set of promoters that normally function in only one direction significantly increases activity in the opposite direction in 67% of cases. Our findings demonstrate that GABP regulates the majority of divergent promoters and suggest that bidirectional transcriptional activity is mediated through GABP binding and transactivation at both divergent and nondivergent promoters.

## Author Summary

Surveys of the locations of genes in the human genome have revealed that a surprising number of genes, greater than 10%, have transcription start sites within 1 kb of one another on opposite strands. These divergent gene pairs, sometimes referred to as bidirectional genes, are common in organisms such as bacteria and yeast, but it is unknown why such an arrangement exists in large, mammalian genomes. Recently, it has become apparent that the promoters of these divergent genes are regulated by a subset of transcription factors, and we have focused on one of these, GA-binding protein (GABP). We find that it regulates a large number of human genes, including the majority of divergent genes, and that its binding is associated with, correlated with, and sufficient for bidirectional transcriptional activity. Although clearly GABP is a major regulator of divergent genes, which carry out a variety of roles critical for the function and survival of the cell, these data also propose novel roles for GABP as a transcription factor. For example, the ability of GABP to promote bidirectional transcription may prove to be biologically relevant in generating many of the transcripts that have been observed outside of protein coding genes.

## Introduction

Since the discovery that more than 10% of the protein-coding genes in the human genome are located on opposite strands with transcription start sites less than 1 kb away from each other, there has been considerable interest in determining why such an arrangement exists [1–3]. Studies in mammals and more distantly related organisms have shown that these divergently transcribed gene pairs have highly correlated expression patterns [3–6]. However, neighboring, nondivergently transcribed genes also often have correlated expression patterns [5,7,8]. This raises the question of whether the regulation of divergent gene pairs is due to some unique aspect of their paired arrangement or merely a consequence of their proximity.

A likely contributor to coregulation of divergent gene pairs is the less than 1-kb-long intergenic region between divergent transcription start sites of an annotated gene on the plus strand and another on the minus strand. Efforts to determine whether differences exist between these promoters and those that do not lie between start sites for two annotated divergent genes (referred to here as general promoters) have yielded mixed results. As a group, divergent promoters exhibit increased colocation with CpG islands, a paucity of TATA elements, and an enriched subset of transcription factor binding sites [3,9]. These features suggest that the functional behavior of divergent promoters is distinct from that of general promoters. Despite these broad differences, when the function of promoters from the two groups was tested by transient transfection of luciferase reporter plasmids into human tissue culture cells, 48% of general promoters were capable of directing transcription in both directions at similar levels, which we refer to as balanced bidirectional activity, compared with 66% of divergent gene promoters [10]. Another study using integrating lentiviral vectors designed for gene therapy demonstrated that several general promoters were capable of transcribing marker genes in both directions, suggesting that general promoters can be capable of bidirectional transcription in a genomic context [11]. This raises the question of whether bidirectional activity is the default state for certain promoters in the absence of directional “repressors.” Despite some distinctive sequence characteristics, no clear functional features differentiate divergent from general promoters.

Our recent analysis of 376 promoters showed that five motifs are over-represented in divergent gene pairs [9]. These motifs correspond to the binding sites of the transcription factors Nrf-1, CCAAT, YY1, Sp1, and GA-binding protein (GABP). GABP, an *ets* family transcription factor, was of particular interest because the presence or absence of its binding site in a set of divergent promoters explained much of the variation in activity observed for a set of reporter deletion constructs [9]. Additionally, the targeted disruption of a GABP site in a 30-bp fragment that we showed directs bidirectional transcription in a luciferase assay abrogated activity in both directions. Analysis of GABP motif-containing promoters from divergent pairs by chromatin immunoprecipitation (ChIP) led us to estimate that up to 57% of such promoters are bound by GABP, suggesting that GABP is an important regulator of divergent gene pairs.

GABP is a key transcriptional regulator of myeloid, cellular respiration (reviewed in [12]), and ribosomal protein genes [13]. GABPα has also been shown to be required for both cell cycle progression [10] and early embryogenesis [14] in mouse. GABP, also known as NRF-2, is unique among *ets*-family transcription factors as it functions as a heterodimer composed of an α and a β subunit. The α subunit, encoded by the GABPA gene, contains the *ets* DNA-binding domain and is widely expressed from a divergent promoter that it self-regulates [15]. The β subunit, encoded by an unrelated gene, GABPB2, contains the transcriptional activation domain as well as four ankyrin repeats necessary for dimerization with the DNA-binding subunit. Tandem GABP sites have been shown to be capable of initiating transcription in the absence of other *cis*-elements [16], indicating that GABP is a potent transcriptional activator. When two recognition sites were present in the same orientation, GABP-initiated transcription was mapped to the more 5′ of the two GABP sites. This property of GABP-dependent transcription, coupled with the observation that many divergent promoters have multiple GABP sites, could explain why overlapping transcription start sites have been observed at some divergent genes [17]. These aspects of GABP biology, along with the presence of one or more of its binding sites in most divergent promoters, suggest that GABP regulates bidirectional transcription.

In this paper, we report our studies of the relationships between GABP, divergent gene promoters, and bidirectional transcriptional activity. First, we used ChIP to measure GABP occupancy at 412 randomly selected promoters from divergent and general genes in three cell types. We then compared binding results for a subset of these promoters in a single cell type to the transient promoter activity in both directions for a representative promoter fragment to test whether binding and bidirectional transcriptional activity are correlated. On the basis of these results, we assayed whether the introduction of a single GABP site into a nondivergent promoter was sufficient for bidirectional transcriptional activity. Our data indicate that GABP not only binds the majority of divergent promoters, but that GABP binding correlates with bidirectional transcriptional activity and that the introduction of a GABP site into a promoter fragment is sufficient for such activity. These findings provide a mechanism for the regulation of bidirectional transcription at divergent and general promoters by GABP.

## Results

### GABP Binds the Majority of Bidirectional Promoters

**Figure 1:**
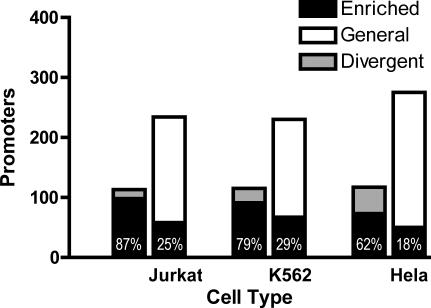
Number and Percentage of Divergent and General Promoters Bound by GABP in Three Different Cell Types Promoters with observed enrichment of 3-fold or more by ChIP-qPCR were considered bound and are depicted as the dark portion of each bar. The majority of divergent promoters are bound by GABP in all three cell types, compared to a much smaller fraction of general promoters.

We performed ChIP followed by quantitative PCR (QPCR) in Jurkat, K562, and HeLa cells with a monoclonal antibody that specifically recognizes GABPα to determine the fraction of divergent and general promoters bound by this factor. We tested for GABP enrichment in ChIP QPCR assays of 121 divergent and 50 general promoters that we previously studied [3]. We also tested 241 experimentally verified, nondivergent promoters identified in the ENCODE Project [18]. We used a cutoff of 3-fold enrichment to classify a promoter as bound; this threshold corresponds to more than three standard deviations from the mean enrichment of five negative control fragments. In all three cell types, a majority of divergent promoters were bound by GABP; 98/113 (87%), 91/115 (79%), and 73/117 (62%) promoters were bound in Jurkat, K562, and HeLa cells, respectively (Figure 1). By comparison, 58/234 (25%), 67/230 (29%), and 50/275 (18%) of general promoters were bound in those same cell types. In all three cell types, the fraction of promoters from divergent genes bound by GABP was significantly higher than for nondivergent genes (*p* < 0.0001 for all cell types; χ^2^). Overall, 105/121 (87%) of the divergent promoters were bound by GABP in at least one cell type, which was significantly greater than 89/291 (31%) general promoters that were bound in at least one cell type (*p* < 0.0001; χ^2^) (Table S1).

As was previously noted [3], divergent promoters contain an increased percentage of CpG dinucleotides relative to general promoters. To exclude the possibility that GABP binds preferentially to CpG-rich promoters and not necessarily to divergent promoters, we compared binding at promoters of divergent and general genes using a subset of our data with similar CpG composition (see Materials and Methods). For divergent promoters, 82/96 (85%), 74/96 (77%), and 61/97 (63%) were bound in Jurkat, K562, and HeLa respectively. For the CpG matched set of general promoters, the fraction bound was 41/96 (43%), 45/96 (47%), and 34/97 (35%). Although the set of general promoters with matched CpG content exhibited a greater fraction of bound promoters than the complete set, divergent promoters still exhibited a significantly greater fraction of promoters bound by GABP (*p* < 0.0001 for all cell types; χ^2^).

**Figure 2:**
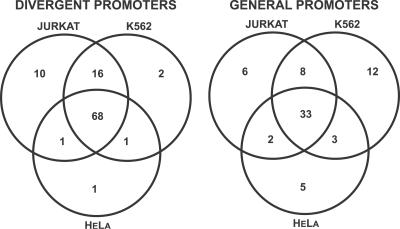
Venn Diagrams Showing the Overlap between GABP-Bound Divergent and General Promoters across Three Cell Types The number of promoters bound in K562 and Jurkat was larger than the intersections of either cell type and HeLa for both promoter classes. The majority of divergent promoters and a smaller fraction of general promoters were bound in all three cell types. The percentage of promoters bound in only a single cell type was larger for general promoters than for divergent promoters. Binding was significantly less cell type specific at divergent promoters.

We also determined whether divergent and general promoters are bound differently by GABP across the tested cell types. We considered only those promoters that were bound in at least one cell type and had binding information for all three cell types. From this set, we then counted the number of promoters bound in all three cell types, in all pairwise combinations, and those that were bound in only single cell types. We then compared the number of promoters bound in each subset of cell lines for divergent promoters and general promoters and found the differences to be statistically significant (*p* = 0.0011; χ^2^) (Table S2). Accordingly, we depict the data for divergent and general promoters as separate Venn diagrams (Figure 2). We found that GABP bound the majority (69%) of divergent promoters in all three cell types. Only 48% of bound general promoters were bound in all three cell types, and the number of general promoters bound in a single cell type was larger than the number of divergent promoters for two of the three cell lines. We conclude from the analysis of three cell lines that general promoters were bound by GABP in a more cell-specific manner, while divergent promoters were more frequently bound in all tested cell types.

Given that *ets*-family transcription factors all recognize very similar binding sites [19], we tested another ubiquitously expressed *ets*-family member, ETS1, by ChIP to exclude the possibility that divergent genes were not regulated more generally by *ets* factors. We tested for ETS1 enrichment at a subset of divergent and general promoters exhibiting a range of GABP enrichment values and cell-line specificity. For divergent promoters, ETS1 bound 19/44 (43%), 3/44 (7%), and 1/47 (2%) of targets in Jurkat, K562, and HeLa cells, respectively, using the same 3-fold enrichment cutoff as was used for GABP (Table S3). For the same targets, GABP bound 33/44 (75%), 25/44 (57%), and 24/47 (51%) promoters in those three cell types. We compared the number of promoters bound or not bound by the two factors independently in each cell type and found that GABP bound significantly more promoters than ETS1 (*p* = 0.0024 for Jurkat, *p* < 0.0001 for K562 and HeLa; χ^2^). We looked for overlap between ETS1 and GABP binding at the same promoter and found 17/44 (37%), 3/44 (7%), and 1/47 (2%) divergent promoters were bound by both factors in the three tested lines. For general promoters, 18/33 (55%), 19/32 (59%), and 18/34 (53%) of selected targets were bound by GABP in the three tested cell lines, compared to 9/33 (27%), 2/32 (6%), and 2/34 (6%) promoters that were bound by ETS1. For all three cell lines, GABP bound a significantly greater number of general promoters than ETS1 (*p* < 0.0001; χ^2^). Despite ETS1 binding to a large number of divergent promoters in Jurkat cells, GABP bound a greater number of targets than ETS1 for all tested cell lines and promoter types.

### GABP Binding Is Associated and Correlated with Bidirectional Transcriptional Activity

A previous study reported that many general promoters are capable of bidirectional transcriptional activity in a transient reporter assay [3]. This finding in combination with our observations on the broad binding distribution of GABP prompted us to explore the relationship between bidirectional transcriptional activity and GABP binding. We used the raw data from 145 promoters tested for luciferase activity in both orientations in the previous study and generated similar data for an additional 88 promoters in HeLa cells. Bidirectional transcriptional activity was previously reported by taking the log_2_ ratio of luciferase activity in the forward direction over the reverse direction (log_2_ Fwd/Rev). As the forward direction was arbitrarily chosen for divergent promoters, we chose to reflect expression activity as the log_2_ ratio of the higher luciferase activity over the lower of the two orientations for a given promoter (log_2_ H/L). All promoters used in this study demonstrated activity above the mean plus three standard deviations of a set of negative controls in at least one direction and no general promoters were observed with reverse activity greater than forward.

We tested for an association between GABP binding and promoter activity in both directions by first classifying each promoter as bound (ChIP enrichment greater than 3-fold) or unbound. We then scored each promoter for whether its representative fragment was capable of directing luciferase activity above background in both directions or only one. We considered all promoters together, regardless of annotation, and compared the number of promoters that were or were not bound and did or did not demonstrate luciferase activity in both directions above background (Table 1). We found that GABP binding and transcriptional activity above background in both directions were highly dependent and statistically significant (*p* < 0.0001; χ^2^). Previously, the majority of divergent promoters were observed to direct luciferase expression to similar levels in both directions (observed log_2_ H/L ratio of less than 2), a phenomenon we call “balanced bidirectional transcription.” To test whether GABP binding and balanced bidirectional activity are associated, we performed another categorical analysis, this time classifying promoter activity as balanced bidirectional or otherwise. For comparison, the percentage of all promoters with a log_2_ H/L ratio of less than 2 was lower than the percentage of promoters with activity above background in both directions (57% versus 80%). For promoters of divergent genes (*n* = 117), 80% exhibited balanced expression while 93% demonstrated promoter activity above background in both directions, compared to 34% and 64% of general promoters (*n* = 116), respectively (Table S4). This more stringent definition of bidirectional activity was also significantly dependent upon GABP binding (*p* = 0.0103; χ^2^). Thus, GABP binding is very strongly associated with transcriptional activity above background in both directions as well as with balanced bidirectional activity.

**Table 1:**
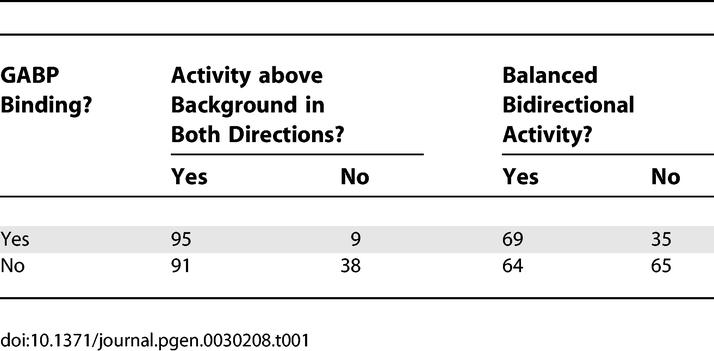
The Number of Promoters That Demonstrated Luciferase Activity above Background in Both Directions or Balanced Bidirectional Activity Subdivided by GABP Binding.

**Figure 3:**
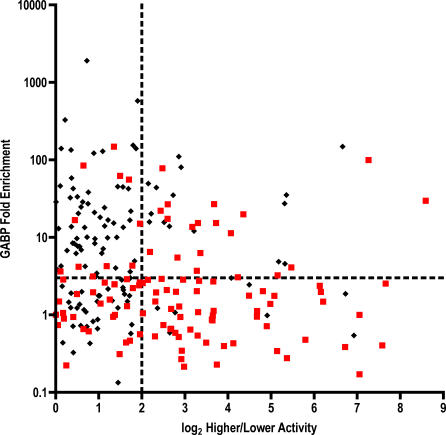
Scatter Plots Depicting the Relationship between GABP ChIP-qPCR Enrichment and Luciferase Activity in HeLa Cells The majority of divergent promoters (black diamonds) was bound by GABP and exhibited a low log_2_ activity ratio. General promoters (red squares) exhibited a much broader distribution of log_2_ H/L expression ratios and lower overall GABP enrichment values. A significant anticorrelation exists between GABP enrichment and log_2_ H/L activity ratios (Spearman's ρ = −0.1773, P = 0.0067), indicating that high GABP enrichment values, corresponding to bound promoters, are associated with low log_2_ promoter activity ratios, typical of balanced bidirectional transcriptional activity. Dotted lines indicate the borders of GABP binding (enrichment of 3-fold or greater) and balanced bidirectional activity (log_2_ H/L less than or equal to 2).

Lastly, we examined the correlation between GABP enrichment at each promoter region and its promoter activity ratio (Figure 3). In addition to high GABP enrichment values (median = 7.12), the majority of divergent promoters (bottom panel) demonstrated low log_2_ H/L ratios (median = 1.02), indicating similar levels of luciferase activity in each direction. General promoters showed a much broader range of expression ratios (median = 2.81), as well as a trend for lower GABP enrichment values (median = 1.66). Across all promoters examined, GABP binding and log_2_ H/L are significantly anticorrelated (Spearman's ρ = −0.1773, *p* = 0.0067). Thus, regardless of the promoter's annotation, promoters bound by GABP (high enrichment values) tended to exhibit balanced bidirectional transcriptional activity (low log_2_ H/L ratio).

### GABP Is Sufficient for Bidirectional Transcriptional Activity

**Figure 4:**
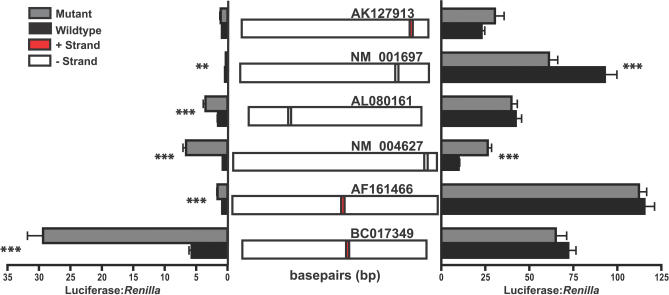
Luciferase Activity in HeLa Cells of General Promoters before and after the Introduction of a Single GABP Site The center of the graph depicts the promoters labeled by their defining cDNA as the cloned sequence used in our assays in 5′ to 3′ orientation on the sense strand for their respective genes with the introduced GABP site, CCGGAAGTG, depicted as bands in either red or white depending on whether the site was introduced in the + or − orientation, respectively. The black bars depict the average luciferase to *Renilla* ratio of the wild-type promoter sequence along with standard error of the mean error bars. The gray bars depict the average promoter activity after the introduction of the GABP site. Asterisks indicate the degree of significance, as determined by Welch's t-test for unequal variances.

Having shown that GABP binding and bidirectional transcriptional activity are correlated, we tested whether the introduction of a GABP site into a promoter is sufficient to induce bidirectional transcriptional activity. We selected six functional promoters that were not bound by GABP in any tested cell type and had little or no expression in the reverse direction, and introduced a single, strict consensus GABP site, CCGGAAGTG, into each promoter through site-directed mutagenesis. Four of six promoters (67%) demonstrated significantly increased luciferase activity in the reverse direction (Figure 4). The average increase in the reverse direction for these promoters was 4.5-fold. At two of these four significantly changed promoters, the increase in reverse activity yielded a log_2_ H/L ratio indicative of balanced bidirectional activity. In contrast, the minimum log_2_ H/L ratio before the introduction of the GABP site was 3.6. Notably, only one of the six promoters demonstrated a significant increase in forward transcriptional activity. Of the two promoters without significant increases in expression, one exhibited significantly decreased expression, while the other was unchanged. In the majority of tested promoters, the addition of a single GABP site was sufficient to increase transcriptional activity in the reverse orientation.

## Discussion

We tested 121 and 291 randomly selected promoters from divergent and general genes, respectively, by ChIP in three cell lines to determine the percentage of genes from each class that were bound by GABP. Using an enrichment threshold of 3-fold for classifying a promoter as bound, we found that 86.8% of divergent promoters and 30.6% of general promoters were bound in at least one cell type. The significantly larger fraction of divergent promoters bound by GABP was also observed when comparing a subset of the two promoter types with matched CpG content. Furthermore, most divergent promoters were bound in all three cell types, suggesting that GABP regulates these promoters in a wide variety of tissues. This is not surprising given the broad expression of GABP and its tendency to regulate ubiquitously and broadly expressed genes, a class to which many divergent gene pairs belong [3,12]. However, a small fraction of divergent genes, as well as a large number of other genes, showed cell line specific binding, so the fraction of promoters regulated by GABP could increase with additional cell type sampling. Collectively, these studies suggest that GABP regulates the vast majority of divergent gene pairs as well as a large number of general genes.

The finding that GABP binds to such a large percentage of divergent gene promoters is surprising, but not unexpected. Our previous study estimated that the transcription factor binds to 57% of divergent gene promoters based upon observed binding frequencies, using a 5-fold enrichment cutoff, for promoters containing a high, medium, or low scoring motif [9]. In this study, we examined a larger number of promoters without first scanning for GABP motifs, tested an additional cell type and used a less conservative but still stringent cutoff. Applying a binding cutoff of 5-fold to our current data decreases the fraction of bound promoters from 87% to 77% for divergent promoters and from 31% to 20% for general promoters; neither change would alter our conclusions. That GABP binds to such a large fraction of both divergent and nondivergent promoters is also not surprising given that its binding site is one of the top ten most common sequence motifs in the human genome [20].

The broad expression of many *ets*-family transcription factors and the similarity in their binding sites led us to test whether ETS1, another ubiquitously expressed family member, might also be involved in the regulation of divergent genes [19]. Interestingly, we found considerable overlap between GABP and ETS1 binding at both divergent and general genes in Jurkat cells. This is perhaps not surprising, as ETS1 is the most highly expressed *ets*-family member in this cell line [19]. However, for all tested cell lines, GABP bound a larger fraction of divergent promoters than did ETS1, and only in Jurkat cells did the latter factor bind more than 10% of targets. In addition to any functional differences conveyed by the binding of these *ets* transcription factors, it would appear that GABP regulates divergent promoters on a widespread basis at a basal level with ETS1 and/or other family members, perhaps serving to modify this activity in a cell-specific manner.

In light of the observation that many general promoters are capable of balanced bidirectional transcriptional activity, we explored the relationship between GABP binding and bidirectional transcriptional activity at all promoters [3]. We found that GABP binding was significantly associated with promoter activity above background in both directions (*p* < 0.0001; χ^2^) and with balanced bidirectional transcription (*p* = 0.0103; χ^2^), supporting a model whereby GABP directs bidirectional transcription. Furthermore, GABP enrichment was significantly anticorrelated with the log_2_ ratio of promoter activity for each direction of the corresponding promoter fragment (Spearman's ρ = −0.1773, *p* = 0.0067). The low correlation coefficient can be partly explained by recognizing that we would not expect a perfect correlation between the degree of enrichment and a lower ratio of activities in each direction. Although the sum of motif occurrence scores correlates with the degree of enrichment [9], there is no reason to expect that the strength or amount of GABP binding would lead to more balanced transcriptional activity. Nonetheless, these data strongly suggest that GABP binding directs bidirectional transcription at divergent and general promoters alike.

Despite strong statistical evidence that GABP binding and bidirectional activity are both correlated and associated, we observed a number of unbound promoters that demonstrated balanced bidirectional activity as well as examples of bound promoters with activity primarily or only in one direction. Many of the former cases likely represent promoters that are bound in vivo but were not scored as bound because they fell below 3-fold enrichment threshold. HeLa cells, in addition to having the lowest percentage of bound divergent promoters that are bound by GABP, had enrichment values on average half that of those observed in K562 and Jurkat cells for those promoters that were bound in all three cell types (unpublished data). In addition, there are almost certainly mechanism(s) for bidirectional activity that are not dependent on GABP binding. For example, five adjacent Sp1 sites were capable of directing bidirectional transcription in vitro [21]. We compared Sp1 and GABP binding at the set of promoters from our previous study [9] and were not able to observe a significant correlation between factor binding in either Jurkat or K562 cells (unpublished data). Nonetheless, it will be interesting to see the degree of overlap between GABP binding and that of Sp1 and other factors with binding sites over-represented among divergent promoters.

There are several possible interpretations for GABP bound promoters that do not conform to our definition of balanced bidirectional transcription. First, not all divergent promoters have log_2_ H/L expression ratios of less than 2. Although this definition of balanced bidirectional transcriptional activity encompasses the majority of divergent promoters, it may exclude many biologically relevant bidirectionally active promoters. In addition, unknown regulatory element(s) may modify the bidirectional activity conveyed by GABP binding. For example, TATA elements are known to convey tissue and start-site specificity and may also influence the directionality of transcription [22]. We tested for a correlation between predicted TATA binding site log-likelihood scores and log_2_ H/L ratio among all promoters in this study and observed a significant, positive relationship (Spearman's ρ = 0.2601, *p* < 0.0001), although, in another study, the introduction of a TATA and/or Inr element into the mouse thymidylate synthase promoter had no effect on its bidirectional activity [23]. In general, it is clear that GABP binding confers bidirectional activity at a large number of promoters, but this activity may be modified in a number of ways on a gene-by-gene basis.

In this study, we observed balanced bidirectional transcriptional activity at 34% of general promoters. One possible explanation for this observation is that a strong promoter contains elements sufficient for leaky expression in the other direction when reversed in our reporter plasmids. To investigate this possibility, we looked for a correlation between activities in each direction for general promoters and found a significant relationship (Spearman's ρ = 0.5883, *p* < 0.0001). However when we compared the luciferase activity values for general promoters that were categorized as having balanced bidirectional activity and those that did not, there was no significant difference (*p* = 0.3419; Mann-Whitney U-test). This suggests that although there may be some expression in the reverse direction due to a strong promoter, this was not sufficient to result in balanced bidirectional activity. Another likely explanation is that the approximately 500-bp promoter fragment we cloned may not have contained all the regulatory sequences necessary for its proper function in the genome. Doubtless other transcription factor(s) and/or boundary elements such as insulators play a role in controlling the level and direction(s) of promoter activity in their endogenous genomic context. Finally, many of the general promoters for which we observed bidirectional activity may actually belong to an unannotated divergent gene pair. A subset of these may prove to be functional bidirectional promoters with a protein-coding gene in one orientation and some other type of transcript in the other. Several recent studies have reported that a much larger percentage of the genome is transcribed than was previously thought, much of this occurring outside of annotated genes [17,24,25], and it will be interesting to see whether these transcripts are in any way specifically initiated or are merely an unintended consequence of GABP regulation of promoter function. While GABP may be responsible for the majority of balanced bidirectional activity, the regulation of directionality at promoters needs more research.

Finally, having shown that GABP is correlated with bidirectional transcriptional activity, we tested whether the introduction of a single GABP site into a promoter with no annotated reverse transcript, little to no promoter activity in the reverse direction and no evidence for GABP binding might be sufficient to induce such activity. Four of six such promoters produced significant increases in reverse luciferase activity after the introduction of a GABP site. Furthermore, for at least one of the promoters that did not show a significant increase in either direction, NM_001697, it is likely that we inadvertently disrupted other sequences necessary for promoter function, as there were significant decreases in activity in both directions. We cannot explain why the introduced site was not capable of directing balanced transcriptional activity in all cases, although it is worth noting that not all divergent promoters exhibit balanced activity. Even in the case of the small change observed in the reverse direction for AF161466, the fold increase was 1.9, which is likely to be biologically relevant for a dosage sensitive gene product. These results are particularly striking given that GABP sites were introduced without regard for site orientation or proximity to the start of transcription. Previous work demonstrated the ability of two tandem GABP sites to drive transcription in either orientation [16], but our study is the first report to our knowledge to show that a single site can drive transcription in both directions in a functional promoter. Given that previous studies have shown that GABP sites might be required for bidirectional transcriptional activity [9,15], these results argue very strongly that a GABP site can be both necessary and sufficient for bidirectional transcriptional activity.

While our data do not suggest how the paired gene arrangement came to exist, they are consistent with a role for GABP regulation in maintaining this relationship. In this model, unknown forces would bring two critical genes together, each with its own promoter, and GABP would reinforce this relationship through its ability to direct balanced, bidirectional transcriptional activity. Given its ability to be sufficient for such activity, the presence of a GABP site could make other factor binding sites redundant over evolutionary time. Thus, the short intergenic region between the gene pair would be free to accumulate mutations, each gene losing its independent regulators and becoming part of an inseparable, truly bidirectional promoter. This supports the conclusion that the promoters of divergent gene pairs do not consist of two overlapping promoters, and that in addition to the previously noted characteristics of these promoters, their bidirectional character is in large part conveyed by GABP binding. Analyses of transcription start site distributions in species from fish to mammals have revealed that although a bimodal distribution, indicative of an abundance of divergent gene pairs, seems to exist only in mammals, 83 divergent pairs have been maintained in proximity and orientation as far separated from humans as *Fugu* [2,4]. A search of HomoloGene (http://www.ncbi.nlm.nih.gov/sites/entrez?db=homologene) indicated that although GABPα has an ortholog in *Drosophila*, the most distant GABPβ ortholog has been observed in Danio rerio (*Fugu* is not currently represented in HomoloGene). The coincident emergence of conserved divergent gene pairs and a GABP heterodimer capable of bidirectional transcriptional activity reinforces the finding that GABP is the major regulator of divergent genes.

In summary, we showed that GABP binds the majority of divergent promoters and is correlated with and sufficient for bidirectional activity. We have established its role as a major regulator of divergent genes, which carry out a variety of activities critical for the function and survival of many different cell types. In addition, GABP binds a large number of nondivergent genes, and further study will be needed to ascertain whether its ability to promote bidirectional transcription genes has widespread biological consequences through the generation of novel and/or noncoding transcripts.

## Materials and Methods

### GABP and ETS1 binding.

ChIP was performed as described [9] in HeLa, K562, and Jurkat cells (ATCC) using either a mouse monoclonal antibody recognizing GABPα, sc-28312, or a polyclonal rabbit antibody against ETS1, sc-350 (Santa Cruz Biotechnology). We tested for GABP enrichment by quantitative PCR of amplicons designed to the promoters of 121 random divergent gene pairs and 50 general genes from a previous study [3], as well as 241 general genes from ENCODE regions [18]. Primers for quantitative PCR as well as corresponding average enrichment values for each target in each cell type can be found in Dataset 1. ETS1 enrichment was assayed for a subset of these targets, 48 divergent and 36 general promoters, using the same primers. Promoters showing greater than 3-fold enrichment relative to the average of five negative controls were considered bound. We then compared the number of GABP bound and unbound promoters for divergent and general promoters for each cell type individually using χ^2^ analysis in the Prism 4.0c (GraphPad Software) software package. We also determined the number of promoters that were bound in at least one cell type and compared this to those that were bound in no cell types for each type of promoter. We also compared the number of promoters that were and were not bound by GABP and ETS1 again for each cell line independently. We considered only promoters with binding information for both factors in each cell type. However, as divergent and general promoters were not randomly sampled for ETS1, we did not compare the fraction of promoters bound for each type of promoter.

### CpG matched promoter set.

We attempted to extract the largest possible set of CpG-content matched divergent and general promoters from our dataset. We calculated CpG percentage based upon the cloned promoter sequence, and then for each cell type, averaged CpG content for promoters for which QPCR data was available. Promoters from each group were then removed until the average CpG content for the two groups was within 0.1% for equal sample sizes. For divergent promoters, the average CpG contents were 31.2%, 31.1%, and 31.0% for Jurkat, K562, and HeLa, respectively. For general promoters, CpG contents were 31.1%, 31.1%, and 31.0% for promoters sampled from those three cell lines.

### Bidirectional promoter activity assay.

The 241 general promoters tested for GABP ChIP enrichment were previously cloned into the plasmid pGL3 Basic (Promega) and verified to be functional promoters as part of the ENCODE project [18]. We amplified these promoters, consisting of the sequence from approximately −500 to +50 bp relative to the start of transcription, by using common primers flanking the MCS (Forward 5′-CATACGCTCTCCATCAAAACAAA-3′, Reverse 5′-TTTATGTTTTTGGCGTCTTCCAT-3′), digested them with *Mlu*I and *Bgl*II (New England BioLabs), and then recloned them into a pGL3 Basic vector with a reversed MCS, yielding a luciferase reporter plasmid with a reversed promoter sequence. A total of 50 ng of each promoter construct was cotransfected with 5 ng of pRLTK (Promega), a *Renilla* luciferase-expressing transfection control, using FuGene (Roche). Transfections were performed in 96-well plates seeded approximately 24 h earlier with 5,000 HeLa cells in each well. Cells were lysed 24 h after transfection and assayed for luminescence on a Victor Light luminometer (Perkin Elmer). The ratio of luciferase to *Renilla* luminescence was calculated for all fragments and compared to a panel of 24 negative controls. Sample ratios that were lower than the mean plus three standard deviations were considered to not be expressed above background and were recorded as 0.1 to permit the calculation of directional activity ratios. Only promoters with activity above background in at least one direction were subjected to further analyses. For 88 promoters we calculated the log_2_ ratio of the promoter orientation with the higher promoter activity over the lower (log_2_ H/L). In all cases, the higher activity of the two orientations corresponded to the sense orientation relative to the annotated gene product. We also calculated log_2_ H/L ratios for 117 divergent and 28 general genes from a previous study [3]. The cloned promoter sequences, luciferase to *Renilla* ratios for each direction and the log_2_ H/L ratio for promoters in this study can be found in Dataset 1. We plotted the activity ratio for each promoter against its corresponding GABP enrichment score and then performed a nonparametric correlation on the data using the Spearman method in the Prism 4.0c software package. We also categorized each promoter as either having luciferase activity above background in one or both promoter orientations. We then compared the number of promoters that were or were not bound by GABP and did or did not demonstrate luciferase activity above background using χ^2^ analysis in Prism 4.0c. We also performed a similar χ^2^ analysis, but this time compared the number of bound and unbound promoters with log_2_ H/L ratios below two, indicating balanced bidirectional activity, or above two.

### Mutagenesis.

We selected six sequences with experimentally confirmed unidirectional activity and no evidence for GABP binding in any cell type. We then designed primers, using the web-based PrimerX tool (http://bioinformatics.org/primerx/), to introduce one to four substitutions around an existing GGAA sequence in the wild-type promoter resulting in a GABP consensus site, CCGGAAGTG. Mutagenesis reactions were performed with Quikchange mutagenesis kit (Stratagene) in accordance with the manufacturer's protocol. The mutant sequences, once confirmed by sequence in each orientation, were then transfected into HeLa cells three times independently in sextuplicate alongside wild-type controls as above. The original cloned promoter sequence, mutagenesis primers, and luciferase to *Renilla* ratios for each direction for mutated and wild-type promoters can be found in Dataset 2. Luciferase to *Renilla* ratios were pooled across the three independent experiments and wild-type ratios were compared to those of the mutant using a two-tailed Welch's t-test in Prism 4.0c.

## Supporting Information

Dataset S1 Table Containing All ChIP and Luciferase Data Presented in This Manuscript along with Cloned Promoter Sequence and ChIP qPCR Primers (516 KB XLS)

Dataset S2 Excel Worksheet Containing Promoter Sequences, Mutagenesis Primers, and Luciferase Data for Nondivergent Promoters into Which a GABP Site Was Inserted (37 KB XLS)

Table S1 The Number of Divergent and General Promoters Bound by GABP in at Least One Cell Type (24 KB DOC)

Table S2 The Number of Divergent and General Promoters Bound by GABP by Cell Type Subset (28 KB DOC)

Table S3 The Number of Divergent and General Promoters Bound by GABP, ETS1, or Both by Cell Type. (28 KB DOC)

Table S4 The Number of Promoters That Demonstrated Luciferase Activity above Background or Balanced Bidirectional as Well as GABP Binding by Promoter Type (32 KB DOC)

### Accession Numbers

The RefSeq (http://www.ncbi.nlm.nih.gov/RefSeq/) accession numbers for the genes and gene products discussed in this paper are: GABPA and ETS1 (NM_002040 and NM_005238, respectively). GABPB2 is listed under accession numbers NM_181427, NM_002041, NM_016655, NM_005254, and NM_016654.

## Abbreviations

ChIP: chromatin immunoprecipitation
GABP: GA-binding protein
QPCR: quantitative PCR
