# Supplementary material for: The ets-Related Transcription Factor GABP Directs Bidirectional Transcription
Source: PLoS Genet. 2007 Nov 16;3(11):e208. doi: 10.1371/journal.pgen.0030208 (PMC2077898; doi:10.1371/journal.pgen.0030208)
Supplement: Supplementary file 1 [file pgen.0030208.st001.doc]

|  | Bound | Unbound |
| --- | --- | --- |
| Divergent | 105 | 16 |
| General | 89 | 202 |
