# Supplementary material for: The ets-Related Transcription Factor GABP Directs Bidirectional Transcription
Source: PLoS Genet. 2007 Nov 16;3(11):e208. doi: 10.1371/journal.pgen.0030208 (PMC2077898; doi:10.1371/journal.pgen.0030208)
Supplement: Supplementary file 2 [file pgen.0030208.st002.doc]

|  | Divergent | General |
| --- | --- | --- |
| Jurkat only | 10 | 6 |
| K562 only | 2 | 12 |
| HeLa only | 1 | 5 |
| Jurkat & K562 | 16 | 8 |
| K562 & HeLa | 1 | 3 |
| Jurkat & HeLa | 1 | 2 |
| Jurkat, K562 & HeLa | 68 | 33 |
