# Supplementary material for: The ets-Related Transcription Factor GABP Directs Bidirectional Transcription
Source: PLoS Genet. 2007 Nov 16;3(11):e208. doi: 10.1371/journal.pgen.0030208 (PMC2077898; doi:10.1371/journal.pgen.0030208)
Supplement: Supplementary file 3 [file pgen.0030208.st003.doc]

| Promoter type | Jurkat (N=44) | | | K562 (N=44) | | | HeLa (N=47) | | |
| --- | --- | --- | --- | --- | --- | --- | --- | --- | --- |
|  | GABP | ETS1 | Both | GABP | ETS1 | Both | GABP | ETS1 | Both |
| Divergent | 33 | 19 | 17 | 25 | 3 | 3 | 24 | 1 | 1 |
| General | 18 | 9 | 8 | 19 | 2 | 2 | 18 | 2 | 2 |
