# Supplementary material for: The ets-Related Transcription Factor GABP Directs Bidirectional Transcription
Source: PLoS Genet. 2007 Nov 16;3(11):e208. doi: 10.1371/journal.pgen.0030208 (PMC2077898; doi:10.1371/journal.pgen.0030208)
Supplement: Supplementary file 4 [file pgen.0030208.st004.doc]

| Promoter Type | GABP binding? | Activity above background? | | Balanced bidirectional activity? | |
| --- | --- | --- | --- | --- | --- |
|  |  | Yes | No | Yes | No |
| Divergent | Yes | 68 | 5 | 58 | 15 |
|  | No | 41 | 3 | 36 | 8 |
| General | Yes | 27 | 4 | 11 | 20 |
|  | No | 50 | 35 | 28 | 57 |
